# Effects of tDCS on Real-Time BCI Detection of Pedaling Motor Imagery

**DOI:** 10.3390/s18041136

**Published:** 2018-04-08

**Authors:** Maria de la Soledad Rodriguez-Ugarte, Eduardo Iáñez, Mario Ortiz-Garcia, José M. Azorín

**Affiliations:** Brain-Machine Interface Systems Lab, Miguel Hernández University of Elche, Avda. de la Universidad S/N Ed. Innova, Elche, 03202 Alicante, Spain; eianez@umh.es (E.I.); mortiz@umh.es (M.O.-G.); jm.azorin@umh.es (J.M.A.)

**Keywords:** transcranial direct current stimulation (tDCS), brain–computer interface (BCI), real-time, pedaling motor imagery, cerebro-cerebellar pathway

## Abstract

The purpose of this work is to strengthen the cortical excitability over the primary motor cortex (M1) and the cerebro-cerebellar pathway by means of a new transcranial direct current stimulation (tDCS) configuration to detect lower limb motor imagery (MI) in real time using two different cognitive neural states: relax and pedaling MI. The anode is located over the primary motor cortex in Cz, and the cathode over the right cerebro-cerebellum. The real-time brain–computer interface (BCI) designed is based on finding, for each electrode selected, the power at the particular frequency where the most difference between the two mental tasks is observed. Electroencephalographic (EEG) electrodes are placed over the brain’s premotor area (PM), M1, supplementary motor area (SMA) and primary somatosensory cortex (S1). A single-blind study is carried out, where fourteen healthy subjects are separated into two groups: sham and active tDCS. Each subject is experimented on for five consecutive days. On all days, the results achieved by the active tDCS group were over 60% in real-time detection accuracy, with a five-day average of 62.6%. The sham group eventually reached those levels of accuracy, but it needed three days of training to do so.

## 1. Introduction

Transcranial direct current stimulation (tDCS) is a modern technique of non-invasive brain stimulation which has the purpose of temporally modulating cortical excitability [1,2]. Currently, its effects are not known with certainty, but they are believed to be dependent on several factors such as intensity applied [3], time of stimulation [4] and size of the electrodes used [5]. The majority of the studies focused their research on applying tDCS to the representation of the upper limbs in the brain to evaluate the performance of the subjects or to improve the quality of life of stroke patients who have had that area affected [6,7,8]. Only relatively few studies attempted to investigate how tDCS could affect the lower limbs [9,10]. This could be due to the challenge of reaching the area of the brain where the legs are represented, which is located deep in the longitudinal fissure corresponding to the primary motor cortex (M1).

From a cognitive perspective, brain activity during a lower limb complex motor task, such as gait or pedaling, involves the supplementary motor area (SMA), M1, the primary somatosensory cortex (S1) and the premotor area (PM) [11,12,13,14]. Moreover, lower limb motor imagery (MI) is also associated with these areas [15]. Hence, if a person imagines a complex motor task, the person will activate a similar neural pathway to that activated when the task is actually being performed. In addition, the cerebellum is a key part during movement coordination, motor learning and cognition [16]. The underlying mechanism of the ascending outputs from the cerebellum relies on sending information to M1 through the dentate nucleus. Some of the axons in this area cross the midline of the brain to terminate in the ventral lateral complex of the thalamus, and then the motor thalamus sends inputs to the M1 and PM areas [17].

On the one hand, research findings have found that tDCS over the cerebellum produces cortical excitability changes in a polarity-specific manner [18]. While cathodal tDCS over the cerebellum decreases the inhibitory tone the cerebellum exerts over M1, anodal tDCS has the opposite effect [19,20]. From a physiological perspective, the principal neuron found in the cortex of the cerebellum is called the Purkinje cell. If the anode is located over the cerebellum, these neurons are excited producing inhibition in the dentate nucleus and resulting in disfacilitation of the motor cortex [21]. On the other hand, cortical excitability over M1 increases when the anode is located over M1 and the cathode over the contralateral hemisphere, or over the contralateral supraorbital region [22,23]. Nevertheless, no research has studied the cerebro-cerebellar pathway where simultaneously the anode is located over M1 and the cathode over the contralateral cerebellum. Doing this could increase the cortical excitability over M1 even more.

Brain–computer interfaces (BCIs) are devices that translate brain waves into commands to control an external device, such as exoskeletons. They can do this, for example, by reading electroencephalographic (EEG) signals from the brain, extracting useful features from those signals, and then using statistical methods to discern between relevant outputs. This technique can improve the rehabilitation process of a person that has suffered a cerebrovascular accident (CVA). The most challenging aspect of using BCIs is to detect neural cognitive processes in real time, so that, as soon as data are received, they are processed. However, researchers usually analyze data offline, where data are studied once the experiment has finished [24,25]. This can produce unrealistic results when compared to a more challenging online analysis, which is more relevant for real-time applications such as rehabilitation therapies involving exoskeletons.

Motor imagery has been detected using EEG-based BCIs in the past, but most studies focused on upper limbs or simple foot movements [26,27,28,29]. Much fewer studies concentrated on lower limb complex tasks such as gait or pedaling [30]. In most of these studies, BCIs have exploited in some way the fact that there is a suppression of the mu waves (8–12 Hz) and beta waves (13–30 Hz) around M1 when a motor task is being imagined [31,32]. The literature involving real-time processing and feedback of BCI signals associated to these types of movements is scarcer, and the methods of reporting results are disperse [26,30,33,34,35,36,37]. Nevertheless, there are many relevant applications of detecting lower limb movement in real time. Indeed, in the long run, it would be desirable to design an online BCI where patients with CVA are rehabilitated with the aid of a lower limb exoskeleton which they are able to control in real time. Additionally, if the effects of tDCS prove to be positive (by exciting M1 and facilitating detection), this could help in improving or simply accelerating the recovery of those patients even more.

Thus, the aim of this work is to strengthen the cortical excitability over M1 and the cerebro-cerebellar pathway by means of a new tDCS configuration to better detect lower limb motor imagery in real time using an online BCI that distinguishes between two different cognitive neural states: relax and pedaling MI. To do that, a single-blind study is carried out where people are randomly divided into two groups, sham and active tDCS, and experimented for five consecutive days. The sham group received a fake stimulation and the active tDCS group was given 0.4 mA. Our hypothesis is that the active tDCS group would achieve better detection accuracy results than the sham group.

## 2. Materials and Methods

### 2.1. Subjects

Fourteen healthy subjects between 23 and 38 years old (26.8 ± 4.9) took part in this experiment (most of them were MSc students). There were twelve male participants and two female participants. All of them were right-footed. None of the subjects had any previous experience with BCIs or MI; they reported no neurological diseases; none of them were medicated; and they were not suffering the consequences of an intoxication during the time the experiments were carried out. Lastly, all participants gave written informed consent according to the Helsinki declaration. The Ethics Committee of the Office for Project Evaluations (Oficina Evaluadora de Proyectos: OEP) of the Miguel Hernández University of Elche (Spain) approved the study.

### 2.2. Experimental Protocol

This section explains the experimental protocol. Several studies which treat different problems such as phantom limb pain, Parkinson’s disease or apraxia of speech after stroke, applied tDCS for five days and reported positive effects [38,39,40]. In addition, a study from [41] stated that the lasting effects of tDCS when it is applied for 15 min were up to 1.5 h. Therefore, taking into account these aspects, our stimulation protocol was established as five consecutive days (Monday to Friday) for 15 min to investigate if there was any improvement in developing pedaling MI.

**Figure 1:**
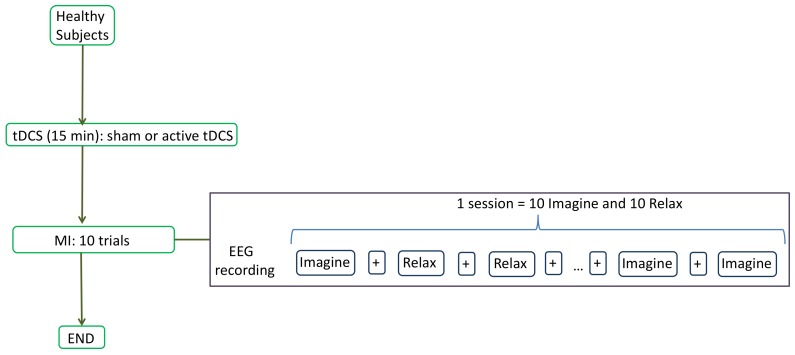
Flow diagram of the experiment for healthy subjects. The subjects were instructed by the screen to perform one of two possible mental tasks: *Relax* or *Imagine*. During *Relax*, subjects had to try not to think about anything, while, during *Imagine*, they had to imagine themselves pedaling. The *Relax* and *Imagine* tasks appeared at random and were always separated by an intermediate period (indicated by the screen with a *+* symbol). The setup also prevented two tasks of the same type to appear more than two times consecutively.

**Figure 2:**
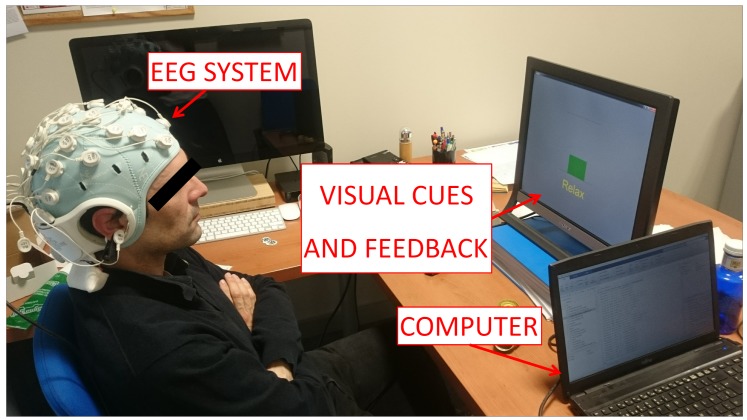
Experimental setup. Subjects sat looking at a screen which fed them with instructions while their EEG signals were recorded. Furthermore, the screen gave feedback about their performance in each task. The participant in the picture gave written informed consent to publish the image.

The experiment consisted on recording the EEG signals (more details on Section 2.3) while the user was performing two mental tasks: relax and imagine. During the imagine task, subjects had to visualize a pedaling movement inside their heads. To remove the placebo effect, a single-blind study was designed in which subjects were randomly divided into two groups: sham or active tDCS. The participants sat in front of a screen which fed them with instructions. Each subject performed 1 session every day which consisted of tDCS supply and MI experiment. First, tDCS (sham or active) was administrated for 15 min (more details in Section 2.4). Then, each subject performed 10 trials of the MI experiment. Each trial included each task (relax and imagine) 10 times. The screen provided three types of instructions: *Relax*, *Imagine* and *+*. *Relax* and *Imagine* tasks lasted 5.8 s and the order appeared at random, but in such a way that no same task appeared more than two times consecutively. This was done to avoid the user to start an expected task beforehand. The symbol *+* was always shown between tasks and lasted 3 s. During *Relax* and *Imagine*, the subjects were told to avoid blinking, swallowing or any other kind of artifacts. They were told to postpone these until the *+* symbol appeared. Figure 1 shows the flow diagram of each session’s experimental protocol, while Figure 2 shows the experimental setup.

The first 4 trials were used to train a SVM classifier with which an online BCI was designed. This is explained in Section 2.5. For the remaining trials, the users received real-time positive feedback about their performance using the output from the BCI. That is, if during the relax task, the BCI detected that the subject had executed mental relaxation, then a green bar increased in size (otherwise it stayed the same size); and similarly with the pedaling MI task. The detection accuracy was calculated for each session, but this information was withheld from the subjects until the end of the last day to avoid influencing them.

### 2.3. EEG Acquisition

The StarStim R32 (Neuroelectrics, Barcelona, Spain) was used to acquire signals from the brain. The device was connected through a USB isolator to the computer. Based on the International 10-10 system, the EEG signals were acquired from 30 channels (P7, P4, CZ, PZ, P3, P8, O1, O2, C2, C4, F4, FP2, FZ, C3, F3, FP1, C1, OZ, PO4, FC6, FC2, AF4, CP6, CP2, CP1, CP5, FC1, FC5, AF3, and PO3) with two reference electrodes (CMS and DRL) at a frequency rate of 500 Hz. The system is shown in Figure 2.

### 2.4. Supply of tDCS

As mentioned before, the idea was to stimulate the cerebro-cerebellar pathway. To do this, a novel montage which aimed at strengthening the neural activity in M1 was proposed. It involved placing the anode over the primary motor cortex in Cz and the cathode over the right cerebro-cerebellum (two centimeters right and one centimeter down of the inion).

**Figure 3:**
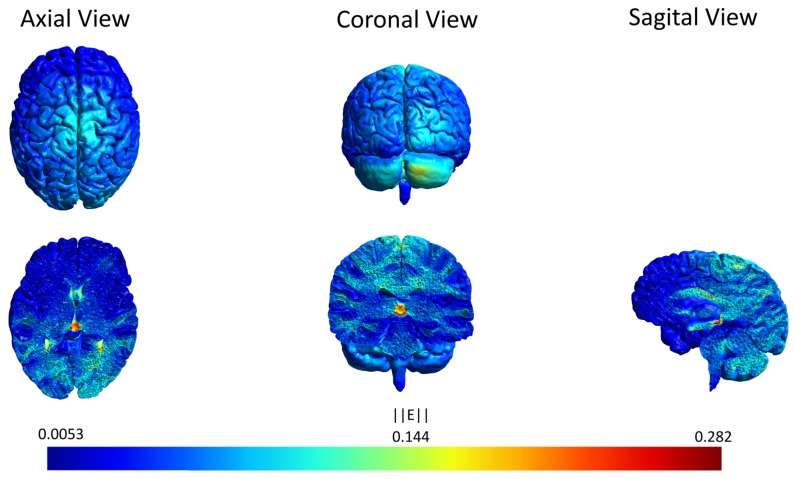
Axial, coronal and sagital view of the tDCS simulation. The scale represents the electric field (V/m) induced by the anode located over Cz and cathode over the right cerebro-cerebellum. The intensity applied was 0.4 mA. The most affected area (red) is close to the red nucleus. The image was generated with SimNIBS.

To corroborate that the cerebro-cerebellar pathway was being stimulated with such a choice of electrode placement, an electric field simulation of the brain was performed first. SimNIBS free platform [42] was used for the simulation, and Figure 3 shows the electric field generated by the anode over Cz (M1) and the cathode over the right cerebro-cerebellum. The parameters were set according to the materials utilized in the experiments. Both electrodes had a radius of 1 cm, 3 mm of thickness and 4 mm of space for the conductive gel. The tDCS intensity chosen was 0.4 mA, which produced 0.127 mA/cm^2^ of current density. This current density was higher than in most studies (roughly 0.06 mA/cm^2^) and it was selected because a previous study reported that a current density of 0.06 mA/cm^2^ was not sufficient to reach the representation of the legs in the brain [43]. The current density also lies inside the range of neurological safety that avoids brain damage [44]. In Figure 3 it can be seen that the most affected area is close to the red nucleus and the thalamus. Both areas belong to the pathway of the ascending outputs from the cerebellum to M1 and PM [45], and therefore we expect this configuration to enhance the excitability in the area of interest.

For the actual experiment, the StartStim R32 supplied anodal tDCS for 15 min at the beginning of each session (one session per day for five consecutive days) through two gel electrodes with a surface area of π cm^2^ (1 cm radius). To create a placebo effect, the sham group received a 3 s ramp up until the intensity chosen, followed by 3 s ramp down; then, no stimulation was provided for almost 15 min until again there was a 3 s ramp up followed by a ramp down. Meanwhile, the active tDCS group received a 3 s ramp up until the intensity chosen, followed by constant stimulation throughout 15 min, and finally a 3 s ramp down.

### 2.5. Brain–Computer Interface (BCI)

As mentioned before, EEG signals were obtained as the subjects performed their relax and pedaling MI tasks. The first two seconds of each task were not considered to avoid influence of the visual cue and assure the total concentration of the subject in the respective task. Signals were processed in 1 s epochs with a 200 ms shift. For each epoch, a 4th order Butterworth high-pass filter with a cut-off frequency of 0.05 Hz was applied to remove the direct current. Then, a Notch filter was used to eliminate the power line interference at 50 Hz. Afterward, a 4th order Butterworth low-pass filter with cut-off frequency of 45 Hz was utilized. Subsequently, based on previous work (e.g., [46,47]), a Laplacian spacial filter was employed as in [48]. This filter eliminates the influence of the other electrodes by means of weighting by their distance. Of these filtered EEG signals, only those coming from nine carefully selected electrodes were considered: Cz, CP1, CP2, C1, C2, C3, C4, FC1 and FC2. These were chosen because the task involved imagination of the lower limbs, so their proximity to the M1, S1, SMA and PM regions of the brain was a deciding factor.

As mentioned above, the first four trials were used to train a support vector machine (SVM) classifier. This classifier is based on hyperplane tasks separation by maximizing the margin between the nearest points of the different tasks [49], with the outcomes obtained using non linear kernels being generally more robust than those of other classifiers [50]. In this work, a radial basis function was used as kernel for the SVM. For every given electrode, the power at each frequency between 6 and 30 Hz (resolution of 1 Hz via Burg’s method) was calculated for each epoch. Then, the powers were separated according to the task (relax or pedaling imagery), normalized and averaged across all task-related epochs of the first four trials. Then, for each electrode, the frequency for which the maximum (normalized) power difference between tasks occurred was chosen and designated as the electrode’s optimal frequency. Lastly, for each epoch, the feature associated to each electrode was the power at the electrode’s optimal frequency, for a total of nine features per epoch. These features were then used to train the SVM classifier.

Therefore, the online BCI designed consisted of filtering the EEG signals of each epoch as described above, finding the nine features (the powers at each electrode’s optimal frequency), and classifying the features with the (already trained) SVM. Thus, for each epoch, the BCI predicted whether it corresponded to a relaxed or pedaling state, and it was able to do this in real time (making a prediction every 0.2 s). The remaining six trials were utilized to determine the performance of the user in the day’s session by measuring the real-time detection accuracy of the online BCI. The real-time detection accuracy was defined as the percentage of total correct classifications divided by the total number of classifications. As mentioned before, real-time positive feedback was given to the user, so that, if the BCI detected a relaxed state while the screen requested *Relax* (a correct classification), a green bar increased in size (otherwise it did not move), and similarly with the pedaling MI task.

## 3. Results

### 3.1. Statistical Analysis

IBM SPSS Statistics 22.0 for Windows (SPSS Inc., Chicago, IL, USA) was used for statistical analysis. First, we wanted to examine the differences in performance between groups (sham and active tDCS). Moreover, we wanted to study, within subjects of each group, the evolution of their performance throughout the five days of the experiment, which we refer to here as plasticity. Therefore, there were two independent variables: group and days; and only one dependent variable: real-time detection accuracy. Thus, a mixed factorial ANOVA was applied, but only after a Mauchly’s test of sphericity was completed to verify the equality of variances of the differences within subjects [51]. In addition, pairwise comparisons between groups for each day, and within subjects of each group between days were computed. For every analysis, a *p*-value less than 0.05 was considered statistically significant.

Table 1 shows the results of applying Mauchly’s test of sphericity. As it can be seen, variances were significantly different (p<0.05), so data violated the sphericity assumption. Consequently, the correction with the biggest power was applied. In this case, it corresponded to Hyunh–Feldt ($\hat{\epsilon }$ = 0.987). After applying this correction, a mixed factorial ANOVA was calculated.

#### 3.1.1. Effects of tDCS in MI

**Figure 4:**
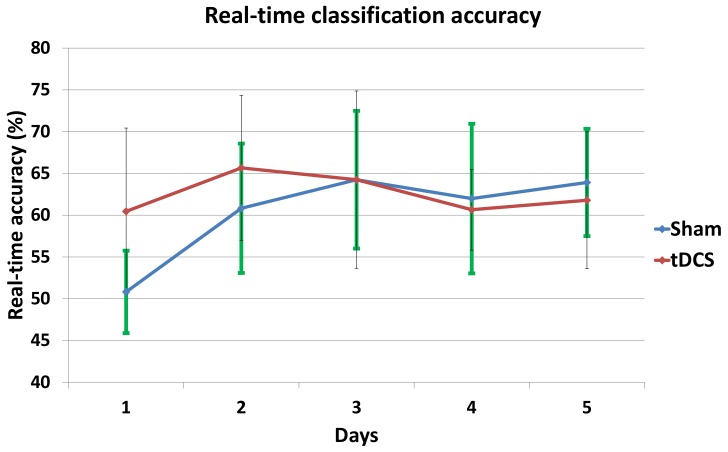
Mean real-time accuracy for all subjects of each group at each day.

Table 2 shows the five-day mean real-time detection accuracies for each subject along with the overall average of the sham and active tDCS groups. In addition, from the mixed factorial ANOVA we obtained that the effects of tDCS in MI were not significant: F(1,12)=0.37, p>0.05, r=0.03. Moreover, Table 3 shows the comparisons, with Bonferroni adjustment applied for multiple comparisons, between both groups for each day. It can be appreciated that for the first day there was a significant difference (p<0.05) in the real-time detection accuracies (see also Figure 4).

#### 3.1.2. MI Plasticity

Figure 4 represents the mean real-time accuracy for each group at each day of the experiment. From the mixed factorial ANOVA, it can be concluded that there was a significant interaction effect between the days and the group of stimulation: F(3.95,47.35)=3.56, p<0.01, r=0.23. Furthermore, Table 4 shows, for each group, the *p*-values comparing day five and the other days. There was only a significant difference between Day 5 and Day 1 within subjects of the sham group (p<0.01).

### 3.2. Optimal Frequencies

The optimal frequencies associated to the BCI model at each electrode on each day are also very useful information. They show where the greatest (normalized) changes in power were occurring, and therefore give a rough idea of the frequency bands that are most important in association with the lower limb motor imagery that is being studied. To present the results of all subjects together, a histogram was made showing, for each day and group, the number of optimal frequencies lying in three important frequency bands: 6–12 Hz (high theta and mu waves), 13–20 Hz (low and mid-range beta waves) and 21–30 Hz (high beta waves). For each group and day, there were a total of 63 optimal frequencies since there were nine electrodes selected for each of the seven subjects in each group. The results are presented in Table 5. The frequency band associated to mu waves seems to be the most preferred.

### 3.3. Real-Time Accuracy and ERD of the Best Subjects

**Figure 5:**
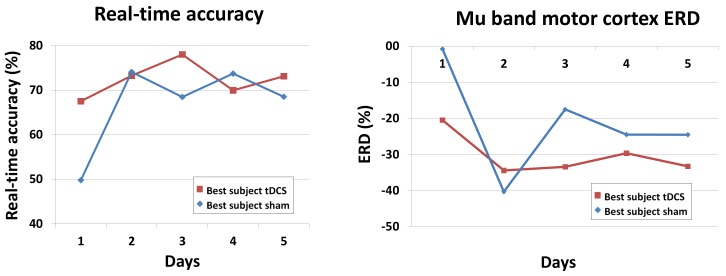
Real-time accuracy and ERD of the best subjects in each group.

As previously mentioned, lower limb motor imagery is thought to be associated to the attenuation of mu and beta waves in M1 [31,32]. This phenomenon is referred to as event-related desynchronization (ERD). To see the changes in ERD, the best subjects of each group were selected based on their five-day real-time detection accuracy (Table 2): Subject 2 of the sham group and Subject 7 of the active tDCS group. Given the results in the previous section and that those electrodes over M1 are thought to be mostly involved, the focus was on the mu waves (8–12 Hz) occurring in the Cz, C1, C2, C3 and C4 electrodes. For an electrode *E*, and for a fixed frequency *f*, the ERD was defined as

(1) $${\mathrm{ERD}}_{E}\left(f\right)=\left(\frac{P(f)-R(f)}{R(f)}\right)\times 100\;,$$

where P(f) is the average of the power at the frequency *f* over all pedaling-epochs, and R(f) is the same but averaged over all relaxing-epochs. Then, the mu band motor cortex ERD for a given day was simply the average of all ${\mathrm{ERD}}_{E}\left(f\right)$ over f=8,9,10,11,12 and E= Cz, C1, C2, C3, C4. These results, along with the real-time accuracies of the two best subjects, are shown in Figure 5.

## 4. Discussion

It can be seen in Table 2, as well as concluded from the mixed factorial ANOVA test, that in general there is no significant difference between the active tDCS and sham groups. Indeed, the active tDCS group achieved 62.6% of real-time detection accuracy and the sham group 60.4%. Nevertheless, the mixed factorial ANOVA results also indicated that there was a significant interaction effect between the days and the groups. This is because the sham and tDCS groups differ significantly in the first day, with the tDCS group having 9.6% better real-time accuracy, and also because within the sham group there is a significant variation when comparing the first and last day of the experiment.

Thus, the results show that the positive effects in performance due to tDCS are only relevant in the first few days, possibly only the first day, as the sham group then adapts and achieves the same performance. This is consistent with a study performed by Fernandez et al. [10] where it was observed that adaptation reached a lower limit the first day due to the simplicity of the task, leading to non-significant differences in the days that followed. However, it contradicts a study by Soekadar et al. [20] on upper limbs which suggested that only after three days did changes due to tDCS started to be differentiated from sham. This could be due to several reasons, including the different location of the tDCS electrodes and their surface area, as well as the different nature of the experiment and the way the data were processed. Longer experiment durations (over five days) could also help to discern the root cause of these slight differences. Meanwhile, the results agree in part with those of Wei et al. [8], which only had a one-day experiment showing slight improvement due to tDCS stimulation. Our results also show such slight improvement in favor of the tDCS group on the first day. Naturally, our real-time accuracy results differed from the offline accuracies shown in the study by Wei et al. [8], since they were able to improve on their classifier offline.

From another point of view, the subjects without stimulation showed evidence of brain plasticity, with an overall improvement of 13% in real-time accuracy from the first to the last day. Hence, their brains seem to have adapted very quickly to the task, meaning that if the intention is to eventually develop a therapy that elapses over several days, perhaps it is not necessary to apply tDCS at all. Having said that, tDCS did show evidence of speeding up improvement in the sense that it seems to have had an instantaneous effect in activating the desired neural pathway. Therefore, the results suggest that the active tDCS immediately induces the maximum performance that a subject could reach and it maintains it each day. Meanwhile, the sham group seems to require two to three days of training to reach the same level as the active tDCS group (see Figure 4). Nevertheless, it should be pointed out that these results involved only healthy subjects. When dealing with patients, we expect to see greater differences between the groups due to the greater potential of improvements in the case of rehabilitating patients.

These conclusions are also corroborated with results in Section 3.2. Indeed, looking at the histogram in Section 5 shows that the optimal frequencies lied the most often in the band containing the mu waves: 6–12 Hz. For the tDCS group on every single day at least 66% of the optimal frequencies lied in this band, while for the sham group from the second day onwards at least 57% of the optimal frequencies were in that band. However, on the first day, only 42% of the optimal frequencies of the sham group where in the preferred frequency band. This seems to indicate that the behavior was more disperse among the frequency bands, and could be a contributing factor explaining why the performance of the sham group in the first day was at chance level, while the tDCS group was already performing better on that day. Namely, it is possible that tDCS favored changes in the mu waves, whereas it took at least one day of training for the sham group to focus the motor imagery on that frequency band.

The results from Section 3.3 also confirm the conclusions, albeit at the level of the best subjects in each group. The best subject in the tDCS group started out with real-time accuracies of nearly 70% and remained above 70% from the second day onwards. On the other hand, the best subject in the sham group started at chance level (50%) and from the second day onwards significantly improved and remained at around 70% accuracy. The mu band motor cortex ERD (8–12 Hz in Cz, C1, C2, C3 and C4) was also interesting. First, as expected, there was presence of ERD each day and for both subjects (negative values because the suppression means that the power while pedaling is lower than when relaxing). On the first day, the sham subject only showed a very subtle ERD, while the tDCS subject had a much more pronounced ERD of around −20%. Then, from the second day onwards, there was an even larger enhancement of the ERD levels of both groups, which remained at an average of about −30%. This shows some level of adaptation of the subjects to the task after the first day.

To address the seemingly low real-time accuracies of around 60% (chance level is 50%), we looked thoroughly at the existing literature to make the appropriate comparisons. It should be noted that the real-time accuracies reported in this study are taking into consideration every single prediction during the experiment (every single epoch is classified). Unfortunately, the current literature on real-time BCIs is somewhat disperse in the way the results are reported [26,30,33,34,35,36,37], but whenever it is possible to compare, our results coincide rather well with those of the literature. Our results are consistent with those of Zich et al. [33] which have a real-time accuracy of 55–65%, [26] with around 65% accuracy (of first 30 sessions), and [30] with around 65% accuracy. Meanwhile, Guger et al. [34] reported the results of the best time point of the best session of each of their three subjects (98%, 93% and 87%), but a careful analysis of their data shows the average real-time classification accuracy is about 80%, 65% and 65% for their three subjects respectively. Prasad et al. [35] averaged the maximum classification accuracies of each task and report them to be 60–75%. All of these studies involve upper limb or simple foot movements with the exception of the study by Liu et al. [30], which involved gait. Therefore, our results actually do not stray far from those found in the literature. Additionally, as pointed out by Prasad et al. [35], these results are reasonable given the fact that all the subjects are novices to BCIs and MI, so their performance is lower than that of experienced users. To improve on the accuracy levels, it might be necessary to change the nature of the motor imagery, since [26] reported significantly better results when doing so. Lastly, in other studies, it was simply not possible to make a fair comparison of the online results [8,36,37].

If the intention is to justify the use of active tDCS over the course of several days or more, then stronger evidence of its effects is needed. In this sense, it could be sensible to change the stimulation montage to one that could possibly lead to more marked differences among the groups. Some possible modifications of the experimental setup would be the number of stimulation anodes and cathodes and their placement, as well as increasing the intensity used whilst keeping safety in mind. From a physiological perspective, we first proposed that the excitation of Purkinje cells in the cerebellum might have the side effect of disfacilitating the motor cortex. However, there is also evidence that their activation can lead to improved motor learning [52]. In fact, anodal stimulation over the cerebellum can speed up learning [19,53,54]. Therefore, we propose an alternative for future use, where two anodes with differing intensities are utilized: one over M1 with relatively high intensity, and another over the cerebro-cerebellum with lower intensity (to prevent any major inhibitory behavior over M1 while still leading to improved motor learning). Meanwhile, a single cathode can be placed in an alternative location (such as FC1 or FC2).

Lastly, it should be said that the online BCI is perfectly apt for use in real-time applications, such as active therapies involving exoskeletons. In the future, we intend to use active tDCS and the online BCI to treat patients that have suffered a CVA accident. The idea is to improve their rehabilitation by engaging them in therapy where they have to control a lower limb exoskeleton in real time.

## 5. Conclusions

In this work, a new tDCS configuration intended to boost the cerebro-cerebellar pathway to improve the detection of lower limb MI via the use of a real-time BCI is tested. One anode is located over M1 and one cathode over the right cerebro-cerebellum. A single-blind experiment with duration of five days is completed using healthy subjects who are randomly separated into two groups: sham and active tDCS. The mental tasks they have to perform are: relax and pedaling MI. The online BCI designed is based on finding the power at an optimal frequency at each of nine carefully selected electrodes in the proximity of M1, S1, PM and SMA. From the very first day, the real-time detection accuracy achieved by the active tDCS group is over 60% and remains around 62.6% on average. However, the sham group needs three days of training to reach that same level of accuracy. This, along with other supporting evidence, indicates possibly that the tDCS has an immediate effect in activating the desired neural pathway, and shows the potential advantages in accelerating recovery of patients undergoing therapy. However, overall, the long-term effects of tDCS seems to have been moderate at best. With this in mind, the stimulation montage could possibly be further improved to increase the effects of tDCS and hopefully justify its use. Lastly, the online BCI designed, with or without tDCS, is a desirable stepping stone in designing therapies that allow recovering patients’ real-time control of lower limb exoskeletons, which is a future endeavor of interest.

## Figures and Tables

**Table 1:** Mauchly’s test of sphericity. Within subjects effect.

					Epsilon	
	Mauchly’s W	df	*p*-Value	Greenhouse-Geisser	Hyunh-Feldt	Lower-Bound
**days**	0.09	9	0.003	0.688	0.987	0.25

**Table 2:** Mean real-time detection accuracy.

Subject	Sham	tDCS
1	61.7	66.6
2	66.9	51.8
3	59.6	55.7
4	64.1	55.9
5	51.5	66.9
6	55.2	68.7
7	63.5	72.4
**Mean**	60.4 ± 5.4	62.6 ± 7.9

**Table 3:** Pairwise accuracy comparison between tDCS and sham group.

Day	1	2	3	4	5
***p*-Value**	0.04	0.29	1.00	0.74	0.60

**Table 4:** Comparison between Day 5 and the rest of the days for each group.

Group	Day	Day	*p*-Value
sham	5	1	0.002
		2	1.00
		3	1.00
		4	1.00
tDCS	5	1	1.00
		2	0.78
		3	0.85
		4	1.00

**Table 5:** Optimal frequencies histogram for each day and group.

Group	Frequency Range	Day 1	Day 2	Day 3	Day 4	Day 5
sham	(6–12) Hz	27	42	52	36	39
	(13–20) Hz	14	8	10	18	5
	(21–30) Hz	22	13	1	9	19
tDCS	(6–12) Hz	42	48	53	49	47
	(13–20) Hz	11	10	5	11	6
	(21–30) Hz	10	5	5	3	10

## Abbreviations

The following abbreviations are used in this manuscript:

CVA	Cerebrovascular accident
MI	Motor imagery
tDCS	Transcranial direct current stimulation
BCI	Brain–computer interface
M1	Primary motor cortex
S1	Primary somatosensory cortex
SMA	Supplementary motor area
PM	Premotor
EEG	Electroencephalographic
SVM	Support vector machine
ERD	Event-related desynchronization
